# Repeated α-GalCer Administration Induces a Type 2 Cytokine-Biased iNKT Cell Response and Exacerbates Atopic Skin Inflammation in Vα14^Tg^ NC/Nga Mice

**DOI:** 10.3390/biomedicines9111619

**Published:** 2021-11-04

**Authors:** Hyun Jung Park, Tae-Cheol Kim, Yun Hoo Park, Sung Won Lee, Jungmin Jeon, Se-Ho Park, Luc Van Kaer, Seokmann Hong

**Affiliations:** 1 Department of Integrative Bioscience and Biotechnology, Institute of Anticancsoer Medicine Development, Sejong University, Seoul 05006, Korea; 0402parkhj@gmail.com (H.J.P.); mitalus1@gmail.com (T.-C.K.); dbsgn703@gmail.com (Y.H.P.); insiderjjang@gmail.com (S.W.L.); jjm4165@gmail.com (J.J.); 2 Department of Life Sciences and Biotechnology, Korea University, Seoul 02841, Korea; sehopark@korea.ac.kr; 3 Department of Pathology, Microbiology and Immunology, Vanderbilt University School of Medicine, Nashville, TN 37232, USA; luc.van.kaer@vumc.org

**Keywords:** atopic dermatitis, NC/Nga mice, iNKT cells, Vα14 TCR transgenic mice, α-GalCer

## Abstract

We have previously shown that Vα14 TCR Tg (Vα14^Tg^) NC/Nga (NC) mice contain increased numbers of double-negative (DN) invariant natural killer T (iNKT) cells that protect against spontaneous development of atopic dermatitis (AD). iNKT cells can regulate immune responses by producing various cytokines such as IFNγ and IL4 rapidly upon stimulation with α-galactosylceramide (α-GalCer), a prototypical iNKT cell agonist. However, the precise role of α-GalCer-activated iNKT cells in AD development remains unclear. Therefore, we examined whether repeated activation of iNKT cells with α-GalCer can regulate the pathogenesis of AD in Vα14^Tg^ NC mice. We found that Vα14^Tg^ NC mice injected repeatedly with α-GalCer display exacerbated AD symptoms (e.g., a higher clinical score, IgE hyperproduction, and increased numbers of splenic mast cells and neutrophils) compared with vehicle-injected Vα14^Tg^ NC mice. Moreover, the severity of AD pathogenesis in α-GalCer-injected Vα14^Tg^ NC mice correlated with increased Th2 cells but reduced Th1 and Foxp3^+^ Treg cells. Furthermore, the resulting alterations in the Th1/Th2 and Treg/Th2 balance were strongly associated with a biased expansion of type 2 cytokine-deviated iNKT cells in α-GalCer-treated Vα14^Tg^ NC mice. Collectively, our results have demonstrated the adverse effect of repeated α-GalCer treatment on skin inflammation mediated by type 2 immunity.

## 1. Introduction

Atopic dermatitis (AD) is an incurable, inflammatory skin disorder characterized by T helper 2 (Th2) cell predominance and IgE hyperproduction [1,2]. Interleukin 4 (IL4) is considered a critical cytokine in the pathogenesis of AD because it induces Th2 cell differentiation, IgE production, and increased Th2 type chemokines such as CCL17 and CCL22 during AD development [3]. IFNγ-mediated Th1 immune responses induced by dendritic cell (DCs)-derived interleukin 12 (IL12) play an important role in regulating Th2-mediated AD [4]. A sustained supply of exogenous IFNγ is effective in preventing the onset of AD in both murine AD models and human patients through modulation of the Th1/Th2 balance [5], implicating IFNγ as a critical target for therapeutic intervention in AD [1]. NC/Nga (NC) mice are widely used to investigate the pathogenesis of AD because these animals exhibit AD-like skin inflammation. NC mice spontaneously develop AD when housed under conventional, but not barrier, conditions. When kept under conventional housing conditions, these animals scratch themselves starting from around eight weeks of age. Consequently, their skin becomes dry, scaly, or flaky, and becomes sensitive to allergens present in conventional housing conditions. The resulting increases in serum IgE and Th2 cells contribute to the pathogenesis of AD [6,7,8].

Invariant natural killer T (iNKT) cells are innate-like T cells that react with glycolipid antigens bound by the major histocompatibility complex (MHC) class I-related protein CD1d. These cells are capable of rapidly producing either anti-inflammatory cytokines (e.g., IL4, IL13, and IL10) or pro-inflammatory cytokines (e.g., IFNγ, TNFα, and IL17) upon stimulation with various stimuli (e.g., cytokines, Toll-like receptor (TLR) ligands, and glycolipids) [9,10]. iNKT cells consist of two subsets (CD4^+^ iNKT or DN iNKT subset), depending on the expression of co-receptors (CD4 and CD8) [11]. While CD4^+^ iNKT cells mainly produce IL4, DN iNKT subset can rapidly produce high amounts of IFNγ, but not IL17, IL4, or IL13 [12]. Since activated iNKT cells interact with various innate/adaptive immune cells (e.g., DCs, macrophages, type 3 innate lymphoid cells (ILC3s), CD4^+^ T cells, CD8^+^ T cells, and regulatory T (Treg) cells), these cells play a pivotal role in either protecting or worsening immune-related diseases even in the absence of exogenous glycolipids [13,14,15]. In addition, interactions between iNKT cells and Treg cells have been implicated in controlling autoimmune diseases [9,15]. Moreover, iNKT cells can enhance the in vivo expansion of Treg cells in the lung and skin, consequently affecting the outcome of type 2 immune diseases such as asthma and AD [13,16].

Alpha-galactosylceramide (α-GalCer), a glycolipid antigen derived from the marine sponge *Agelas mauritianus*, can selectively activate iNKT cells in a CD1d-dependent manner [17]. Thus, activation of iNKT cells by a single injection of α-GalCer at early time points of a disease process can contribute to the onset of inflammatory diseases [18,19]. Moreover, repeated stimulation of iNKT cells with α-GalCer affects the outcome of a variety of diseases. For example, repeated α-GalCer treatment of R6/2 Tg mice accelerates the severity of Huntington’s disease pathogenesis via increased infiltration of CD69^+^ iNKT cells into the brain [20]. In addition, repeated in vivo α-GalCer stimulation plays a pro-tumorigenic role by inducing anti-inflammatory IL10-producing iNKT cells [20,21,22]. Furthermore, such stimulation can promote a substantial increase in anergic (i.e., hyporesponsive) iNKT cells, thereby alleviating symptoms of experimental lupus nephritis [20,21,22].

We have previously demonstrated that overexpression of iNKT cells in Vα14^Tg^ NC mice effectively prevents the development of AD via a concurrent increase in Th1 and Treg cells [13]. In this study, we investigated whether repeated α-GalCer administration affects the pathogenesis of AD in Vα14^Tg^ NC mice. Further, we examined the effects of this repeated iNKT cell activation on CD4^+^ T cell polarization during AD development.

## 2. Materials and Methods

### 2.1. Study Design

This study was designed to determine the effect of α-GalCer on the pathogenesis of AD in Vα14^Tg^ NC mice. To address this issue, Vα14^Tg^ NC mice were intraperitoneally (i.p.) injected with α-GalCer. Subsequently, splenocytes and serum were harvested and further analyzed by flow cytometry and ELISA.

### 2.2. Mice

Wild-type (WT) B6 and NC mice were purchased from Jung Ang Lab Animal Inc. Vα14^Tg^ mice were provided by Dr. A. Bendelac (University of Chicago, Chicago, IL, USA). Vα14^Tg^ B6 mice were backcrossed to NC mice for more than eleven generations to obtain Vα14^Tg^ NC mice. These mice were maintained at Sejong University and were used for experiments at 6–12 weeks of age. They were maintained on a 12 h light/12 h dark cycle in a temperature-controlled barrier facility with free access to food and water. Mice were fed a γ-irradiated sterile diet and provided with autoclaved tap water. For monitoring spontaneous AD development, mice were transferred at six weeks of age from the barrier facility to the conventional animal facility. Age- and sex-matched mice were used for all experiments. The animal experiments were approved by the Institutional Animal Care and Use Committee of Sejong University (SJ-20190301E1).

### 2.3. Cell Isolation and Culture

Splenic CD4^+^ T cells were isolated from Vα14^Tg^ NC mice using a magnetically activated cell sorting (MACS) system (Miltenyi Biotec, Bergisch Gladbach, Germany), following the manufacturer’s instructions [23]. CD4^+^ T cells were enriched >96% after MACS. Primary cells were cultured in RPMI 1640 (Gibco BRL, Grand Island, NY, USA) culture media supplemented with 10% FBS, 10 mM HEPES, 2 mM L-glutamine, 100 units/mL penicillin-streptomycin, and 5 μM 2-mercaptoethanol.

### 2.4. Flow Cytometry

The following monoclonal antibodies (mAbs) were obtained from BD Biosciences (San Jose, CA, USA): fluorescein isothiocyanate (FITC)-, phycoerythrin (PE)-Cy7- or allophycocyanin (APC)-conjugated anti-CD3ε (clone 145-2C11); FITC-, PE-Cy7, or APC-conjugated anti-CD4 (clone RM4–5); PE-conjugated anti-CD103 (clone M290); PE-Cy7-conjugated anti-GITR (clone DTA-1); PE-Cy7-conjugated anti-CD11b (clone M1/70); APC-conjugated anti-CD25 (clone PC61); PE-conjugated anti-IL10 (clone JES5-16E3); PE-Cy7-conjugated anti-CD117 (c-kit) (clone 2B8); FITC- or PE-conjugated anti-Foxp3 (clone NRRF-30); PE-conjugated anti-IL17A (clone eBio17B7). The following mAbs from Thermo Fisher Scientific were used: FITC- or APC-conjugated anti-CD19 (clone ID3); FITC- or APC-conjugated anti-Ly-6G (Gr-1) (clone RB6-8C5). FITC- or PE-conjugated anti-IL4 (clone BVD6-24G2); FITC- or APC-conjugated anti-FcεRI (clone MAR-1); PE-conjugated anti-IFNγ (clone XMG1.2). Cells were harvested and washed twice with cold 0.5% BSA-containing PBS (FACS buffer) for staining surface markers. For blocking Fc receptors, the cells were incubated with anti-CD16/CD32 mAbs (clone 2.4G2) on ice for 10 min and subsequently stained with fluorescently labeled mAbs. Flow cytometric data were acquired using a FACSCalibur flow cytometer (Becton Dickson, San Jose, CA, USA) and analyzed using FlowJo software (Tree Star Inc., Ashland, OR, USA).

### 2.5. Intracellular Cytokine Staining

For intracellular staining, splenocytes were incubated with brefeldin A, an intracellular protein transport inhibitor (10 μg/mL), in RPMI medium for 2 h at 37 °C. The cells were stained for cell surface markers, fixed with 1% paraformaldehyde, washed once with cold FACS buffer, and permeabilized with 0.5% saponin. The permeabilized cells were then stained for an additional 30 min at room temperature with the indicated mAbs (PE-conjugated anti-IFNγ, anti-IL4, anti-IL17, anti-IL10, or PE-conjugated isotype control rat IgG mAbs). Fixation and permeabilization were performed using a Foxp3 staining kit (eBioscience, San Diego, CA, USA) with the indicated mAbs (PE-conjugated anti-Foxp3, or isotype control rat IgG mAbs). More than 5000 cells per sample were acquired using a FACSCalibur, and the data were analyzed using the FlowJo software package (Tree Star Inc., Ashland, OR, USA).

### 2.6. ELISA

Serum IgE levels were measured with a sandwich ELISA (clone R35-72 for capturing IgE and R35-118 for detecting IgE; BD PharMingen, San Jose, CA, USA). The optical density was measured at 450 nm with an immunoreader (Bio-Tek ELX-800, Winooski, VT, USA).

### 2.7. Analysis of Skin Sections

The dorsal skins were fixed in 4% paraformaldehyde, embedded in paraffin, and cut into 6 μm sections using a microtome (RM 2235, Leica, Wetzlar, Germany). The sections were then stained with hematoxylin and eosin (H&E) to analyze histological changes. The cells were counted with a microscope at a magnification of 400 times. The cell density was expressed as the total number of cells in ten high-power fields (400×) for each section.

### 2.8. Scoring the Severity of Skin Lesions

Skin lesions were scored at the indicated time points. The scoring was based on the severity of lichenification, edema, erosion/excoriation, scarring/dryness, and erythema/hemorrhage. The total clinical skin severity score was defined as the sum of the five signs (none = 0; mild = 1; moderate = 2; and severe = 3).

### 2.9. Intraperitoneal Injection of α-GalCer into Mice

Alpha-GalCer was purchased from Enzo Life Sciences (Farmingdale, NY, USA). Vα14^Tg^ NC mice were transferred to conventional housing conditions at six weeks of age to develop AD spontaneously, and these mice were i.p. injected with α-GalCer (2 μg) dissolved in PBS once per week from 6 weeks of age for a total of 12 weeks. Littermate Vα14^Tg^ NC mice injected with PBS were used as negative controls. These mice were measured for clinical AD signs once a week from 6 weeks of age for a total of 12 weeks.

### 2.10. Statistical Analysis

Statistical significance was determined using Excel (Microsoft, Redmond, WA, USA). Student’s *t*-test was performed for the comparison of two groups (* *p* < 0.05, ** *p* < 0.01, and *** *p* < 0.001 were considered significant in the Student’s *t*-test). Two-way ANOVA analysis was carried out using VassarStats (http://vassarstats.net/anova2u.html) (accessed on 11 January 2021) (^#^ *p* < 0.05, ^##^ *p* < 0.01, and ^###^ *p* < 0.001 were considered to be significant in the two-way ANOVA).

## 3. Results

### 3.1. Vα14^Tg^ NC Mice Become Susceptible to AD upon Repeated α-GalCer Treatment

**Figure 1:**
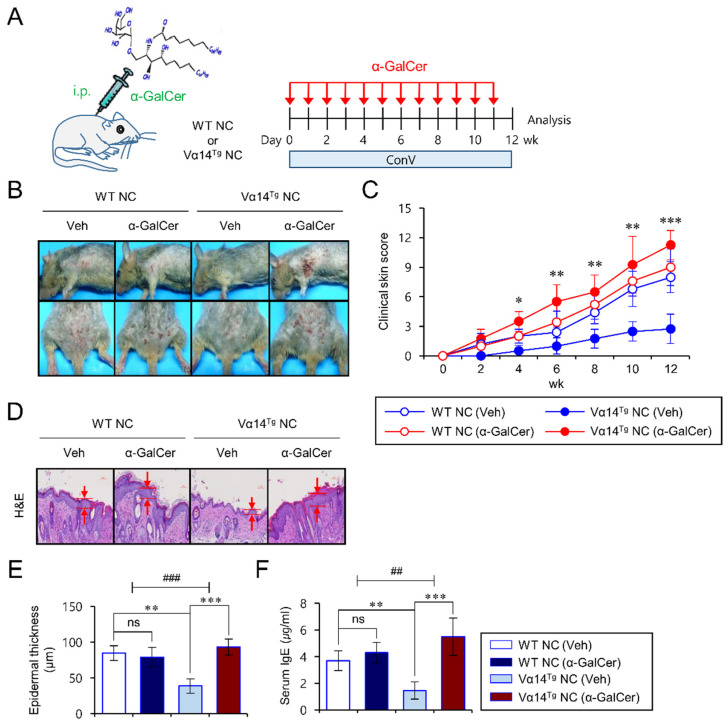
Vα14^Tg^ NC mice become susceptible to AD upon repeated α-GalCer treatment. (**A**) WT NC and Vα14^Tg^ NC mice were i.p. injected with either Veh (*n* = 4) or α-GalCer (2 μg; *n* = 4) once per week from 6 weeks of age for a total of 12 weeks under conventional housing conditions to spontaneously develop AD. All the samples were prepared from mice at 12 weeks post transfer to conventional housing conditions for AD development. (**B**,**C**) The clinical symptoms were measured once a week to monitor the onset of AD. (**D**,**E**) The skins were prepared from WT NC or Vα14^Tg^ NC mice. (**D**) Skin lesions were sectioned and stained with H&E. (**E**) The epidermal thickness was measured in 10 random high-power fields (400×) per sampled lesion. (**F**) Serum IgE levels were measured by ELISA. The mean values ± SD (*n* = 4–5 in A, B, C, D, E, and F; per group in the experiment; Student’s *t*-test; * *p* < 0.05, ** *p* < 0.01, *** *p* < 0.001) are shown. Two-way ANOVA (Vα14 TCR Tg × α-GalCer) showed an interaction between these two factors. One representative experiment of two experiments is shown (^##^ *p* < 0.01 and ^###^ *p* < 0.001).

We have previously shown that overexpression of iNKT cells in Vα14^Tg^ NC mice protects these animals from AD by suppressing skin inflammation and IgE hyperproduction [13]. Moreover, the absence of CD1d-dependent iNKT cells unleashed AD in Vα14^Tg^ NC mice, supporting the protective role of iNKT cells in AD development [13]. Thus, we wanted to determine whether activation of iNKT cells can impact the pathogenesis of AD in Vα14^Tg^ NC mice. The glycolipid α-GalCer, a well-known iNKT cell agonist, was employed to assess this question. During spontaneous development of AD under conventional animal housing conditions, Vα14^Tg^ NC mice were in vivo administrated with α-GalCer once a week starting from 7 weeks of age. At the same time, these mice were monitored for clinical parameters, including clinical score, epidermal thickness, and total serum IgE level compared with control groups of mice (Figure 1A). Consistent with our previous study, we confirmed that vehicle (Veh)-injected Vα14^Tg^ NC mice exhibited significantly lower levels of skin inflammation compared with Veh-injected WT NC mice. In contrast, α-GalCer-injected Vα14^Tg^ NC mice showed progressive AD-like symptoms, including increased clinical scores, epidermal thickness, and total IgE levels, compared with Veh-injected Vα14^Tg^ NC mice and WT NC mice (Figure 1B–F). However, when housed under specific pathogen-free (SPF) conditions, repeated α-GalCer activation did not have any influence on the outcome of AD pathogenesis in either WT NC or Vα14^Tg^ NC mice (Figure S1). This result implies that α-GalCer itself does not initiate allergic immune responses in the absence of AD-inducing environmental factors (i.e., allergens provided most likely by the conventional housing conditions) in NC mice. Together, these results suggest that repeated treatment with α-GalCer diminishes AD-preventive properties of iNKT cells in Vα14^Tg^ NC mice, resulting in aggravated AD pathogenesis.

### 3.2. Repeated In Vivo α-GalCer Treatment Biases iNKT Cells of Vα14^Tg^ NC Mice towards Type 2 Cytokine Production

**Figure 2:**
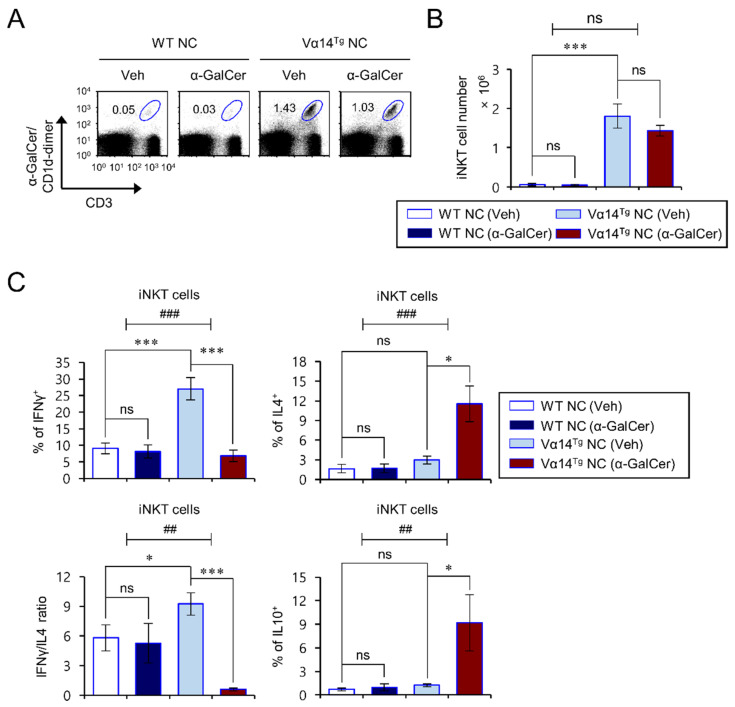
Repeated in vivo α-GalCer treatment changes the iNKT cell phenotype of Vα14^Tg^ NC mice towards type 2 cytokine production. (**A**–**C**) Spleens were prepared from mice as shown in Figure 1. (**A**,**B**) The frequency and cell number of splenic iNKT cells (α-GalCer/CD1d-dimer^+^CD3^+^) were determined by flow cytometry. (**C**) Intracellular IFNγ, IL4, and IL10 were analyzed in splenic iNKT cells (α-GalCer/CD1d-dimer^+^CD3^+^). The mean values ± SD (*n* = 4 in (**A**–**C**); per group in the experiment; Student’s *t*-test; * *p* < 0.05 and *** *p* < 0.001) are shown. Two-way ANOVA (Vα14 TCR Tg × α-GalCer) showed an interaction between these two factors. One representative experiment out of two experiments is shown (^##^ *p* < 0.01 and ^###^ *p* < 0.001).

It has been reported that activated iNKT cells can rapidly release large amounts of type 1 cytokines (IFNγ, IL2) and type 2 cytokines (IL4, IL10). Furthermore, IFNγ and IL4 derived from iNKT cells are known to exert distinct effects on allergic disorders. For example, iNKT cell-derived IFNγ showed a protective effect against Th2-related diseases such as asthma, whereas iNKT cell-derived IL4 increased the pathogenicity of asthma [24,25]. Since a cytokine imbalance in the IFNγ/IL4 ratio from iNKT cells is associated with the pathogenesis of allergic diseases, we determined whether repeated α-GalCer activation alters the cytokine profiles of activated iNKT cells from Vα14^Tg^ NC mice. To test this issue, iNKT cell cytokine profiles were examined in the spleen from α-GalCer-treated Vα14^Tg^ and littermate WT NC mice after maintaining these animals for 12 weeks under conventional housing conditions where spontaneous AD develops. The ratio of IFNγ/IL4 cytokine production from splenic iNKT cells was markedly decreased by repeated α-GalCer treatment in Vα14^Tg^ NC mice (Figure 2A–C). Moreover, the IL4- and IL10-producing cell frequency among iNKT cells in the spleen was significantly higher in α-GalCer-injected Vα14^Tg^ NC mice than in Veh-injected Vα14^Tg^ NC mice (Figure 2C). However, we did not find any significant differences in the number and cytokine production of splenic iNKT cells between α-GalCer-injected and Veh-injected littermate WT NC mice, which might be due to the relative paucity of iNKT cells in WT NC mice owing to a defect in the Vβ8 TCR gene segment. Moreover, this altered cytokine profile of iNKT cells induced following repeated α-GalCer injection was also seen when the animals were housed under SPF conditions. Similar to the conventional housing conditions, we found an increase in IL4- and IL10-producing iNKT cells but a decrease in IFNγ-producing iNKT cells in the spleen of α-GalCer-injected Vα14^Tg^ NC mice housed under SPF conditions (Figure S2). Taken together, these results indicate that repeated in vivo injection of α-GalCer into Vα14^Tg^ NC mice biases the response of iNKT cells from a type 1 towards a type 2 cytokine production profile.

### 3.3. Alpha-GalCer-Mediated Exacerbation of AD Pathogenesis Correlates with a Reduced Th1/Th2 Cytokine Ratio of CD4^+^ T Cells in Vα14^Tg^ NC Mice

**Figure 3:**
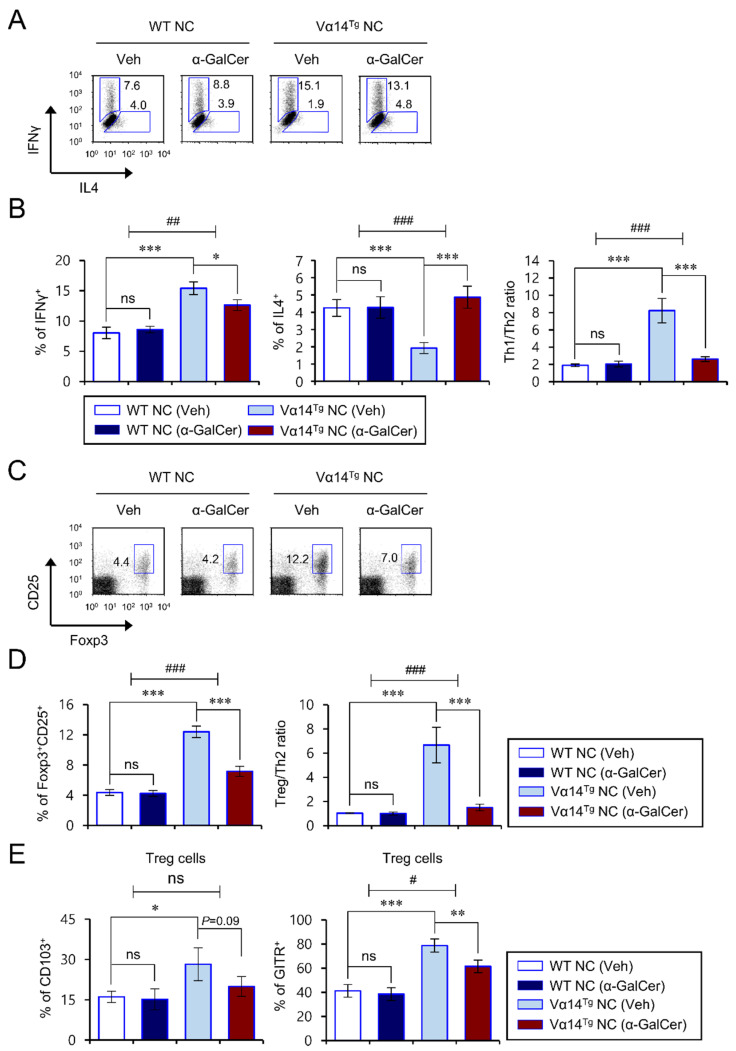
α-GalCer-mediated exacerbation of AD pathogenesis correlates with reduced Th1/Th2 cytokine ratios and Treg cells in Vα14^Tg^ NC mice. (**A**–**E**) Spleens were prepared from mice as shown in Figure 1. (**A**,**B**) IFNγ- and IL4-producing populations in splenic CD4^+^ T cells were determined by flow cytometry. (**C**,**D**) The percentage of Foxp3^+^CD25^+^ cells among CD4^+^ T cells was evaluated by flow cytometry. (**A**,**C**) Representative FACS plots. (**B**,**D**) Summary of data. (**E**) Cell surface expression of CD103 and GITR on splenic Foxp3^+^CD25^+^ Treg cells was analyzed by flow cytometry. The mean values ± SD (*n* = 4 in (**A**–**E**); per group in the experiment; Student’s *t*-test; * *p* < 0.05, ** *p* < 0.01, *** *p* < 0.001) are shown. Two-way ANOVA (Vα14 TCR Tg × α-GalCer) showed an interaction between these two factors. One representative experiment of two experiments is shown (^#^ *p* < 0.05, ^##^ *p* < 0.01 and ^###^ *p* < 0.001).

Low expression levels of the cytokine IL12 can profoundly bias immune responses towards Th2 dominance, as seen in patients with AD [26,27]. Therefore, to test whether iNKT cell polarization by repeated α-GalCer injection affects CD4^+^ T cell differentiation, we measured the IFNγ and IL4 cytokine expression patterns of CD4^+^ T cells from α-GalCer-treated Vα14^Tg^ NC mice with advanced AD. Consistent with our previous report, CD4^+^ T cells from Veh-injected Vα14^Tg^ NC mice produced high levels of Th1 but low levels of Th2 cytokines compared with Veh-injected WT NC mice (Figure 3A,B). However, repeated α-GalCer injection into Vα14^Tg^ NC mice caused a decrease in Th1 cells but an increase in Th2 cells, ultimately resulting in dramatically reduced Th1/Th2 ratios among splenic CD4^+^ T cells (Figure 3A,B). In contrast, such changes were not observed for the frequencies of Th1 or Th2 populations among CD4^+^ T cells between Veh- and α-GalCer-injected WT NC mice (Figure 3A,B).

### 3.4. Repeated α-GalCer Treatment Contracts Splenic Treg Cells in Vα14^Tg^ NC Mice

Treg cells play essential roles in regulating immune responses. When Treg cells become dysregulated, pathogenic Th2 cells and serum IgE levels are drastically elevated in allergic diseases such as AD. In particular, a Treg/Th2 imbalance highly correlates with the severity of AD pathogenesis [1,28,29]. Thus, we wondered whether repeated α-GalCer activation might affect the generation of Treg cells in Vα14^Tg^ and littermate WT NC mice during AD development. To address this issue, we measured the frequency of Treg cells in the spleen after repeated α-GalCer treatment. We found that splenic Treg cells in Veh-injected Vα14^Tg^ NC mice were approximately 200% higher than in Veh-injected WT NC mice. However, repeated α-GalCer-injection into Vα14^Tg^ NC mice statistically decreased the prevalence of splenic Treg cells by 40% compared with Veh-injected Vα14^Tg^ NC mice, indicating that the ratio of Treg cells to Th2 cells was markedly reduced in α-GalCer-injected Vα14^Tg^ NC mice (Figure 3C,D). We next examined the effect of repeated α-GalCer treatment on Treg-associated molecules (CD103 and GITR) of the splenic Treg populations in Vα14^Tg^ NC mice. Alpha-GalCer injection resulted in a modest decrease in CD103 expression but a significant decline in GITR expression among splenic Treg cells in Vα14^Tg^ NC mice (Figure 3E), which implies that repeated α-GalCer injection may downregulate Treg cell function as well as its frequency. However, in WT NC mice, neither Treg cell number nor Treg-associated molecule expression were significantly affected by α-GalCer injection. These findings collectively suggest that α-GalCer-stimulated iNKT cells downregulate Treg cells quantitatively and qualitatively in Vα14^Tg^ NC mice, which likely contributes to the spontaneous development of AD.

### 3.5. Repeated α-GalCer-Injection Expands Splenic Mast Cells and Neutrophils in Vα14^Tg^ NC Mice

**Figure 4:**
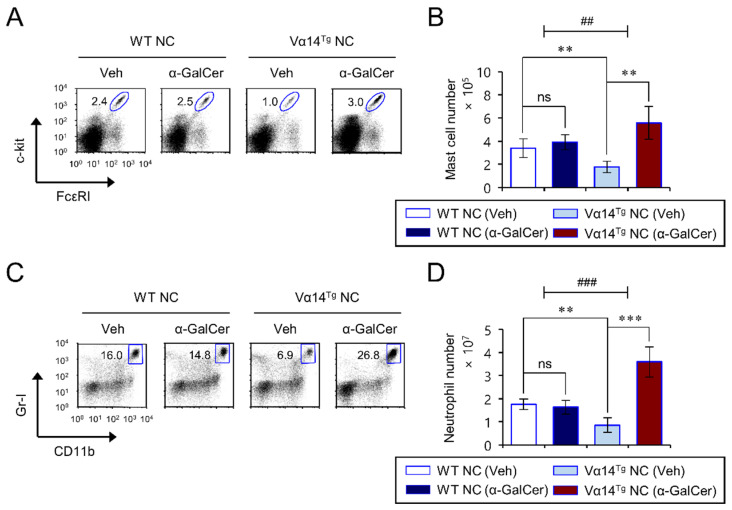
Repeated α-GalCer-injection expands splenic mast cells and neutrophils in Vα14^Tg^ NC mice. Spleens were prepared from mice as shown in Figure 1. (**A**,**B**) The frequency (**A**) and cell number (**B**) of mast cells (FcεRI^+^c-kit^+^CD3^−^CD19^−^) were determined by flow cytometry. (**C**,**D**) The frequency (**C**) and cell number (**D**) of neutrophils (CD11b^+^Gr-1^+^ CD3^−^CD19^−^) were determined by flow cytometry. The mean values ± SD (*n* = 4 in (**A**–**D**); per group in the experiment; Student’s *t*-test; ** *p* < 0.01, *** *p* < 0.001) are shown. Two-way ANOVA (Vα14 TCR Tg × α-GalCer) showed an interaction between these two factors. One representative experiment of two experiments is shown (^##^ *p* < 0.01, and ^###^ *p* < 0.001).

An increase in mast cells and their capacity to be activated by IgE antibodies contributes to the pathogenesis of AD [1]. In addition, neutrophil accumulation is essential for turning skin inflammation into a chronic condition [30]. Moreover, mast cell-derived histamine and neutrophil-derived CXCL10 stimulate histamine receptors and chemokine receptor CXCR3, respectively, on sensory neurons to drive itch in AD skin lesions [31,32]. Thus, we explored whether repeated α-GalCer injection induces phenotypic changes in splenic mast cells and neutrophils in Vα14^Tg^ NC mice during spontaneous AD progression. We found that α-GalCer treatment induces a significant increase in mast cells and neutrophils in Vα14^Tg^ NC mice (Figure 4A–D). Furthermore, consistent with our previous report, Vα14^Tg^ NC mice displayed substantially lower splenic mast cells and neutrophils than WT littermate NC mice (Figure 4A–D).

## 4. Discussion

The present study has demonstrated that repeated α-GalCer treatment enhances allergic immune responses in Vα14^Tg^ NC mice. Furthermore, repeated α-GalCer treatment polarizes iNKT cells towards type 2 cytokine production in Vα14^Tg^ NC mice, resulting in enhanced Th2 immune responses followed by expansion of mast cells and neutrophils, a hallmark of AD progression.

Previous studies are consistent with the possibility that exposure to endogenous or exogenous glycolipids can play either protective or pathogenic roles in AD development through phenotypic changes in iNKT cells. For example, endogenous lipids (e.g., β-D-glucopyranosylceramide and iGb3) can accumulate in DCs during bacterial infection and subsequently be interpreted as innate danger signals to activate both mouse and human iNKT cells [33,34]. These studies indicate that TLR stimulation mediated by microbial infection (e.g., *Staphylococcus aureus*) can affect inflammatory immune responses by activating iNKT cells. In addition, emerging evidence suggests a role of foreign glycolipids in the regulation of immune response via iNKT cell stimulation. For example, house dust extracts and the fungus (*Aspergillus fumigatus*)-derived glycosphingolipid, asperamide B, directly activate iNKT cells in a CD1d-dependent manner, ultimately resulting in asthma exacerbation [35,36]. These studies indicate that repeated exposure to AD-inducing environmental factors (e.g., microbial glycolipids) can induce either anergic (IL10^high^) or type 2 cytokine-deviated iNKT cells (IL4^high^ and IFNγ^low^) in a CD1d-dependent manner. However, glycolipids derived from *Helicobacter pylori* affect the expansion and activation of type 1 cytokine-biased iNKT cells, which consequently promote Th1-biased cytokine responses [16]. In addition, glycolipids derived from commensal bacteria can activate hepatic iNKT cells to produce inflammatory cytokines such as IFNγ, resulting in concanavalin A-induced liver injury [37]. Furthermore, intestinal bacteria such as *Sphingomonas* species carrying iNKT cell antigens induce the full maturation of iNKT cells, indicating that intestinal microbes affect the phenotype and functions of iNKT cells in mice through TCR-dependent and TLR-independent mechanisms [38]. Interestingly, it has been reported that treatment with CD1d-binding lipid antagonists (e.g., α-lactosylceramide (α-LacCer) and di-palmitoyl-phosphatidylethanolamine polyethylene glycol (DPPE-PEG)) can protect against α-GalCer-induced airway hyperreactivity [39,40]. These previous studies suggest that accelerated AD development by exposure to glycolipid antigens could be blocked by CD1d-binding lipid antagonists. In this regard, it will be worthwhile to further investigate whether iNKT antagonist treatment can regulate the progression of AD in α-GalCer-treated Vα14^Tg^ NC mice.

Moreover, a previous study demonstrated that oral administration of α-GalCer selectively activates IL4-producing iNKT cells through pSTAT6 upregulation in the MLN [41]. However, in contrast to conventional conditions, repeated α-GalCer treatment under SPF conditions failed to induce AD in Vα14^Tg^ NC mice (Figure S1). This result implies that allergens responsible for initiating skin inflammation should be present. Thus, in the SPF environment, even in the face of a type 2 cytokine bias in iNKT cells, Th2-type skin inflammation fails to develop in the absence of antigens or foreign antigens that can initiate dermatitis.

Although it is generally thought that mast cells contribute to the pathogenesis of AD [42], inhibitory effects of mast cells on AD have also been reported [43,44]. For example, the absence of mast cells exacerbates the spontaneous development of allergic skin inflammation in constitutively active STAT6 Tg mice, which develop spontaneous allergic inflammation [43]. In addition, mast cell-derived IL2 suppresses chronic allergic dermatitis by increasing Treg cells [44]. Based on these previous studies, the role of mast cells in the pathogenesis of AD is still controversial. Thus, in future studies, it will be informative to further investigate whether mast cells are critical effectors in eliciting AD pathogenesis through the generation of mast cell-deficient Vα14^Tg^ NC mice, by backcrossing Sash^−/−^ mice onto the NC genetic background.

It has been previously demonstrated that activated iNKT cells promote an increase in Treg population through IL2-dependent mechanisms [45]. Moreover, activated iNKT cells increased not only Foxp3 expression but also the suppressive functions on Treg cells following recognition of glycolipids such as bacteria-derived diacylglycerols; consequently, iNKT cell-primed Treg cells suppressed IL4 but not IFNγ production by iNKT cells [46]. Functional alterations in iNKT subpopulations have been reported in AD patients. For example, there is a selective decrease in CD4^−^ [47] and DN [48] iNKT cells, or an increase in CD4^+^ iNKT cells [49] in AD patients. Moreover, our previous study demonstrated that DN iNKT cells from Vα14^Tg^ NC mice enhanced the frequency and functions of splenic Treg cells in the skin, resulting in protection against the onset of AD [13]. Furthermore, DN iNKT cells protect against airway hyperreactivity by expanding allergen-specific Foxp3^+^ Treg cells [12,16]. Our recent finding also demonstrated that high levels of IL2 production by Vα14^Tg^ NC-derived DN iNKT cells induce the expansion of Treg cells, resulting in restraint of AD development [13]. However, we found that Foxp3^+^ Treg cells are significantly reduced in the spleen of α-GalCer-treated Vα14^Tg^ NC mice. Since it has previously been reported that IL4 treatment inhibits TGFβ-mediated Treg cell differentiation in a STAT6-dependent manner [50], enhanced IL4-producing iNKT cells in α-GalCer-injected Vα14^Tg^ NC mice may account for this decrease of Treg cells, ultimately contributing to AD pathogenesis.

## 5. Conclusions

In conclusion, our results provide evidence that long-term exposure to glycolipids such as α-GalCer is associated with an increased incidence of AD. Furthermore, the expansion of IL4-producing iNKT cells contributes to an increased Th2-type immune response in α-GalCer-injected Vα14^Tg^ NC mice, resulting in Th1/Th2 imbalance under conventional housing conditions. Therefore, long-term glycolipid administration regimens for immunotherapeutic purposes should consider phenotypic alterations in iNKT cells that may cause undesired side effects.

## Data Availability

The data are available from the corresponding author on reasonable request.

## Supplementary Materials

The following are available online at https://www.mdpi.com/article/10.3390/biomedicines9111619/s1, Figure S1: Repeated α-GalCer treatment fails to induce significant AD pathogenesis in Vα14^Tg^ NC mice under SPF conditions, Figure S2: Repeated α-GalCer treatment polarizes Vα14^Tg^ NC iNKT cells toward type 2 cytokine production under SPF conditions.

## References

[1] Park H.J., Lee S.W., Hong S. (2018). Regulation of Allergic Immune Responses by Microbial Metabolites. Immune Netw..

[2] Lee S.W., Park H.J., Jeon J., Park Y.H., Kim T.C., Jeon S.H., Seong R.H., Van Kaer L., Hong S. (2021). Ubiquitous Overexpression of Chromatin Remodeling Factor SRG3 Exacerbates Atopic Dermatitis in NC/Nga Mice by Enhancing Th2 Immune Responses. Int. J. Mol. Sci..

[3] Furue M. (2020). Regulation of Skin Barrier Function via Competition between AHR Axis versus IL-13/IL-4JAKSTAT6/STAT3 Axis: Pathogenic and Therapeutic Implications in Atopic Dermatitis. J. Clin. Med..

[4] Weidinger S., Beck L.A., Bieber T., Kabashima K., Irvine A.D. (2018). Atopic dermatitis. Nat. Rev. Dis. Primers.

[5] Hattori K., Nishikawa M., Watcharanurak K., Ikoma A., Kabashima K., Toyota H., Takahashi Y., Takahashi R., Watanabe Y., Takakura Y. (2010). Sustained exogenous expression of therapeutic levels of IFN-gamma ameliorates atopic dermatitis in NC/Nga mice via Th1 polarization. J. Immunol..

[6] Jin H., He R., Oyoshi M., Geha R.S. (2009). Animal models of atopic dermatitis. J. Investig. Dermatol..

[7] Takano N., Arai I., Kurachi M. (2003). Analysis of the spontaneous scratching behavior by NC/Nga mice: A possible approach to evaluate antipruritics for subjects with atopic dermatitis. Eur. J. Pharmacol..

[8] Vestergaard C., Yoneyama H., Murai M., Nakamura K., Tamaki K., Terashima Y., Imai T., Yoshie O., Irimura T., Mizutani H. (1999). Overproduction of Th2-specific chemokines in NC/Nga mice exhibiting atopic dermatitis-like lesions. J. Clin. Investig..

[9] Van Kaer L., Wu L. (2018). Therapeutic Potential of Invariant Natural Killer T Cells in Autoimmunity. Front. Immunol..

[10] Yang G., Driver J.P., Van Kaer L. (2018). The Role of Autophagy in iNKT Cell Development. Front. Immunol..

[11] Kumar A., Suryadevara N., Hill T.M., Bezbradica J.S., Van Kaer L., Joyce S. (2017). Natural Killer T Cells: An Ecological Evolutionary Developmental Biology Perspective. Front. Immunol..

[12] Chuang Y.T., Leung K., Chang Y.J., DeKruyff R.H., Savage P.B., Cruse R., Benoit C., Elewaut D., Baumgarth N., Umetsu D.T. (2019). A natural killer T-cell subset that protects against airway hyperreactivity. J. Allergy Clin. Immunol..

[13] Park H.J., Lee S.W., Park S.H., Van Kaer L., Hong S. (2020). Selective Expansion of Double Negative iNKT Cells Inhibits the Development of Atopic Dermatitis in Valpha14 TCR Transgenic NC/Nga Mice by Increasing Memory-Type CD8(+) T and Regulatory CD4(+) T Cells. J. Investig. Dermatol..

[14] Park H.J., Lee S.W., Van Kaer L., Hong S. (2021). CD1d-Dependent iNKT Cells Control DSS-Induced Colitis in a Mouse Model of IFNgamma-Mediated Hyperinflammation by Increasing IL22-Secreting ILC3 Cells. Int. J. Mol. Sci..

[15] Lee S.W., Park H.J., Cheon J.H., Wu L., Van Kaer L., Hong S. (2018). iNKT Cells Suppress Pathogenic NK1.1(+)CD8(+) T Cells in DSS-Induced Colitis. Front. Immunol..

[16] Chang Y.J., Kim H.Y., Albacker L.A., Lee H.H., Baumgarth N., Akira S., Savage P.B., Endo S., Yamamura T., Maaskant J. (2011). Influenza infection in suckling mice expands an NKT cell subset that protects against airway hyperreactivity. J. Clin. Investig..

[17] Morita M., Motoki K., Akimoto K., Natori T., Sakai T., Sawa E., Yamaji K., Koezuka Y., Kobayashi E., Fukushima H. (1995). Structure-activity relationship of alpha-galactosylceramides against B16-bearing mice. J. Med. Chem..

[18] Wang H., Feng D., Park O., Yin S., Gao B. (2013). Invariant NKT cell activation induces neutrophil accumulation and hepatitis: Opposite regulation by IL-4 and IFN-gamma. Hepatology.

[19] Lee S.W., Park H.J., Van Kaer L., Hong S., Hong S. (2018). Graphene oxide polarizes iNKT cells for production of TGFbeta and attenuates inflammation in an iNKT cell-mediated sepsis model. Sci. Rep..

[20] Park H.J., Lee S.W., Im W., Kim M., Van Kaer L., Hong S. (2019). iNKT Cell Activation Exacerbates the Development of Huntington’s Disease in R6/2 Transgenic Mice. Mediat. Inflamm..

[21] Sag D., Krause P., Hedrick C.C., Kronenberg M., Wingender G. (2014). IL-10-producing NKT10 cells are a distinct regulatory invariant NKT cell subset. J. Clin. Investig..

[22] Uchida T., Nakashima H., Yamagata A., Ito S., Ishikiriyama T., Nakashima M., Seki S., Kumagai H., Oshima N. (2018). Repeated administration of alpha-galactosylceramide ameliorates experimental lupus nephritis in mice. Sci. Rep..

[23] Ju A., Lee S.W., Lee Y.E., Han K.C., Kim J.C., Shin S.C., Park H.J., EunKyeong Kim E., Hong S., Jang M. (2019). A carrier-free multiplexed gene editing system applicable for suspension cells. Biomaterials.

[24] Hachem P., Lisbonne M., Michel M.L., Diem S., Roongapinun S., Lefort J., Marchal G., Herbelin A., Askenase P.W., Dy M. (2005). Alpha-galactosylceramide-induced iNKT cells suppress experimental allergic asthma in sensitized mice: Role of IFN-gamma. Eur. J. Immunol..

[25] Gaspar-Elsas M.I., Queto T., Masid-de-Brito D., Vieira B.M., de Luca B., Cunha F.Q., Xavier-Elsas P. (2015). alpha-Galactosylceramide suppresses murine eosinophil production through interferon-gamma-dependent induction of NO synthase and CD95. Br. J. Pharmacol..

[26] Takahashi N., Akahoshi M., Matsuda A., Ebe K., Inomata N., Obara K., Hirota T., Nakashima K., Shimizu M., Tamari M. (2005). Association of the IL12RB1 promoter polymorphisms with increased risk of atopic dermatitis and other allergic phenotypes. Hum. Mol. Genet..

[27] Itazawa T., Adachi Y., Okabe Y., Hamamichi M., Adachi Y.S., Toyoda M., Morohashi M., Miyawaki T. (2003). Developmental changes in interleukin-12-producing ability by monocytes and their relevance to allergic diseases. Clin. Exp. Allergy.

[28] Fyhrquist N., Lehtimaki S., Lahl K., Savinko T., Lappetelainen A.M., Sparwasser T., Wolff H., Lauerma A., Alenius H. (2012). Foxp3+ cells control Th2 responses in a murine model of atopic dermatitis. J. Investig. Dermatol..

[29] Kalekar L.A., Rosenblum M.D. (2019). Regulatory T cells in inflammatory skin disease: From mice to humans. Int. Immunol..

[30] Oyoshi M.K., He R., Li Y., Mondal S., Yoon J., Afshar R., Chen M., Lee D.M., Luo H.R., Luster A.D. (2012). Leukotriene B4-driven neutrophil recruitment to the skin is essential for allergic skin inflammation. Immunity.

[31] Voisin T., Chiu I.M. (2018). Molecular link between itch and atopic dermatitis. Proc. Natl. Acad. Sci. USA.

[32] Walsh C.M., Hill R.Z., Schwendinger-Schreck J., Deguine J., Brock E.C., Kucirek N., Rifi Z., Wei J., Gronert K., Brem R.B. (2019). Neutrophils promote CXCR3-dependent itch in the development of atopic dermatitis. Elife.

[33] Brennan P.J., Tatituri R.V., Brigl M., Kim E.Y., Tuli A., Sanderson J.P., Gadola S.D., Hsu F.F., Besra G.S., Brenner M.B. (2011). Invariant natural killer T cells recognize lipid self antigen induced by microbial danger signals. Nat. Immunol..

[34] Darmoise A., Teneberg S., Bouzonville L., Brady R.O., Beck M., Kaufmann S.H., Winau F. (2010). Lysosomal alpha-galactosidase controls the generation of self lipid antigens for natural killer T cells. Immunity.

[35] Wingender G., Rogers P., Batzer G., Lee M.S., Bai D., Pei B., Khurana A., Kronenberg M., Horner A.A. (2011). Invariant NKT cells are required for airway inflammation induced by environmental antigens. J. Exp. Med..

[36] Albacker L.A., Chaudhary V., Chang Y.J., Kim H.Y., Chuang Y.T., Pichavant M., DeKruyff R.H., Savage P.B., Umetsu D.T. (2013). Invariant natural killer T cells recognize a fungal glycosphingolipid that can induce airway hyperreactivity. Nat. Med..

[37] Wei Y., Zeng B., Chen J., Cui G., Lu C., Wu W., Yang J., Wei H., Xue R., Bai L. (2016). Enterogenous bacterial glycolipids are required for the generation of natural killer T cells mediated liver injury. Sci. Rep..

[38] Wingender G., Stepniak D., Krebs P., Lin L., McBride S., Wei B., Braun J., Mazmanian S.K., Kronenberg M. (2012). Intestinal microbes affect phenotypes and functions of invariant natural killer T cells in mice. Gastroenterology.

[39] Lai A.C., Chi P.Y., Thio C.L., Han Y.C., Kao H.N., Hsieh H.W., Gervay-Hague J., Chang Y.J. (2019). alpha-Lactosylceramide Protects Against iNKT-Mediated Murine Airway Hyperreactivity and Liver Injury Through Competitive Inhibition of Cd1d Binding. Front. Chem..

[40] Lombardi V., Stock P., Singh A.K., Kerzerho J., Yang W., Sullivan B.A., Li X., Shiratsuchi T., Hnatiuk N.E., Howell A.R. (2010). A CD1d-dependent antagonist inhibits the activation of invariant NKT cells and prevents development of allergen-induced airway hyperreactivity. J. Immunol..

[41] Lee Y.J., Wang H., Starrett G.J., Phuong V., Jameson S.C., Hogquist K.A. (2015). Tissue-Specific Distribution of iNKT Cells Impacts Their Cytokine Response. Immunity.

[42] Wedman P.A., Aladhami A., Chumanevich A.P., Fuseler J.W., Oskeritzian C.A. (2018). Mast cells and sphingosine-1-phosphate underlie prelesional remodeling in a mouse model of eczema. Allergy.

[43] Sehra S., Serezani A.P.M., Ocana J.A., Travers J.B., Kaplan M.H. (2016). Mast Cells Regulate Epidermal Barrier Function and the Development of Allergic Skin Inflammation. J. Investig. Dermatol..

[44] Hershko A.Y., Suzuki R., Charles N., Alvarez-Errico D., Sargent J.L., Laurence A., Rivera J. (2011). Mast cell interleukin-2 production contributes to suppression of chronic allergic dermatitis. Immunity.

[45] Li W., Ji F., Zhang Y., Wang Y., Yang N., Ge H., Wang F. (2008). Cooperation of invariant NKT cells and CD4+CD25+ T regulatory cells in prevention of autoimmune diabetes in non-obese diabetic mice treated with alpha-galactosylceramide. Acta Biochim. Biophys. Sin..

[46] Venken K., Decruy T., Aspeslagh S., Van Calenbergh S., Lambrecht B.N., Elewaut D. (2013). Bacterial CD1d-restricted glycolipids induce IL-10 production by human regulatory T cells upon cross-talk with invariant NKT cells. J. Immunol..

[47] Magnan A., Mely L., Prato S., Vervloet D., Romagne F., Camilla C., Necker A., Casano B., Montero-Jullian F., Fert V. (2000). Relationships between natural T cells, atopy, IgE levels, and IL-4 production. Allergy.

[48] Oishi Y., Sakamoto A., Kurasawa K., Nakajima H., Nakao A., Nakagawa N., Tanabe E., Saito Y., Iwamoto I. (2000). CD4-CD8- T cells bearing invariant Valpha24JalphaQ TCR alpha-chain are decreased in patients with atopic diseases. Clin. Exp. Immunol..

[49] Takahashi T., Nakamura K., Chiba S., Kanda Y., Tamaki K., Hirai H. (2003). V alpha 24+ natural killer T cells are markedly decreased in atopic dermatitis patients. Hum. Immunol..

[50] Dardalhon V., Awasthi A., Kwon H., Galileos G., Gao W., Sobel R.A., Mitsdoerffer M., Strom T.B., Elyaman W., Ho I.C. (2008). IL-4 inhibits TGF-beta-induced Foxp3+ T cells and, together with TGF-beta, generates IL-9+ IL-10+ Foxp3(−) effector T cells. Nat. Immunol..

