# Supplementary material for: Repeated α-GalCer Administration Induces a Type 2 Cytokine-Biased iNKT Cell Response and Exacerbates Atopic Skin Inflammation in Vα14Tg NC/Nga Mice
Source: Biomedicines. 2021 Nov 4;9(11):1619. doi: 10.3390/biomedicines9111619 (PMC8615984; doi:10.3390/biomedicines9111619)
Supplement: Supplementary file 1 [file biomedicines-09-01619-s001.zip › biomedicines-1375500-supplementary.pdf]

Supporting documents to:

Repeated α-GalCer administration induces a type 2 cytokine-biased iNKT cell response and exacerbates atopic skin inflammation in Vα14^{Tg} NC/Nga mice

This document includes:

-Supplementary figures 1-2

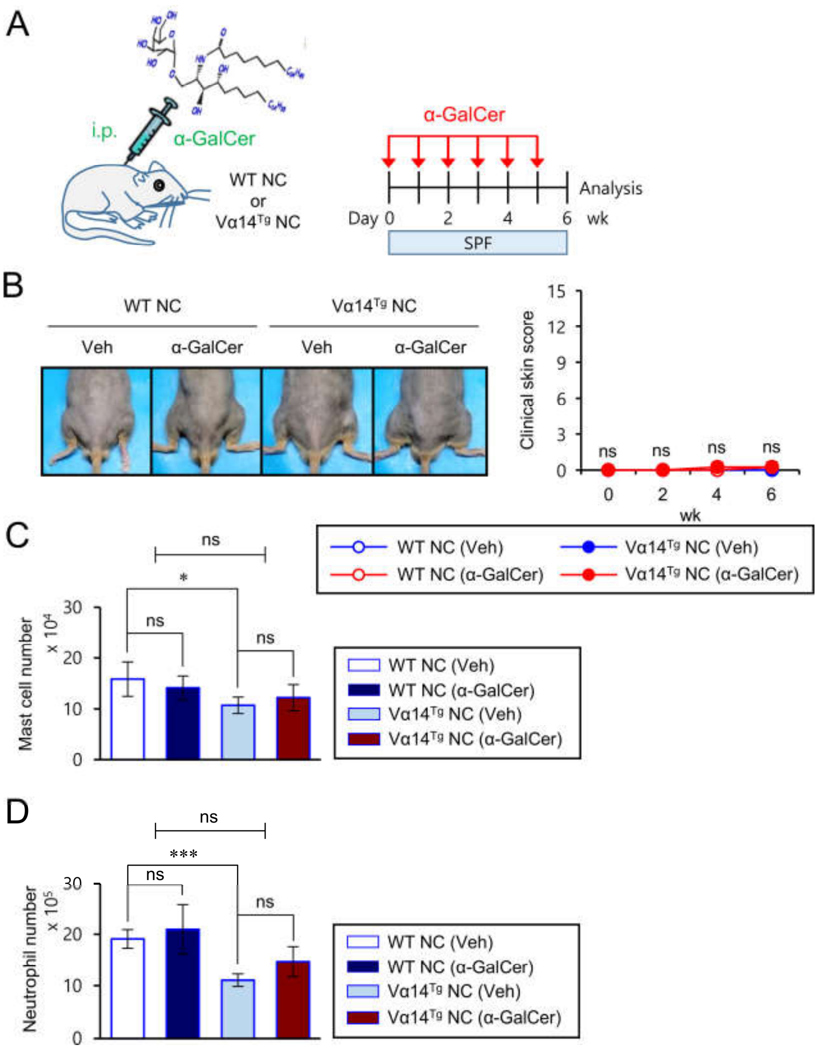

Figure S1. Repeated α-GalCer treatment fails to induce significant AD pathogenesis in Vα14^{Tg} NC mice under SPF conditions.

(A) Both WT NC and Vα14^{Tg} NC mice were i.p. injected with either Veh (n = 4) or α-GalCer (2 μg; n = 4) once a week from 6 weeks of age for a total of 6 weeks under SPF conditions to spontaneously develop AD. (B) The clinical symptoms were measured once a week to monitor the onset of AD. (C-D) Spleens were prepared from mice, as shown in Fig. S1A. The absolute cell numbers of (C) mast cells (FcεRI^{+}c-kit^{+}CD3^{-}CD19^{-}) and (D) neutrophils (CD11b^{+}Gr-1^{+} CD3^{-}CD19^{-}) were determined by flow cytometry. The mean values ± SD (n = 4 in A, B, C, and D; per group in the experiment; Student's t-test; * $P < 0.05$, *** $P < 0.001$) are shown. Two-way ANOVA (Vα14 TCR Tg × α-GalCer) showed an interaction between these two factors.

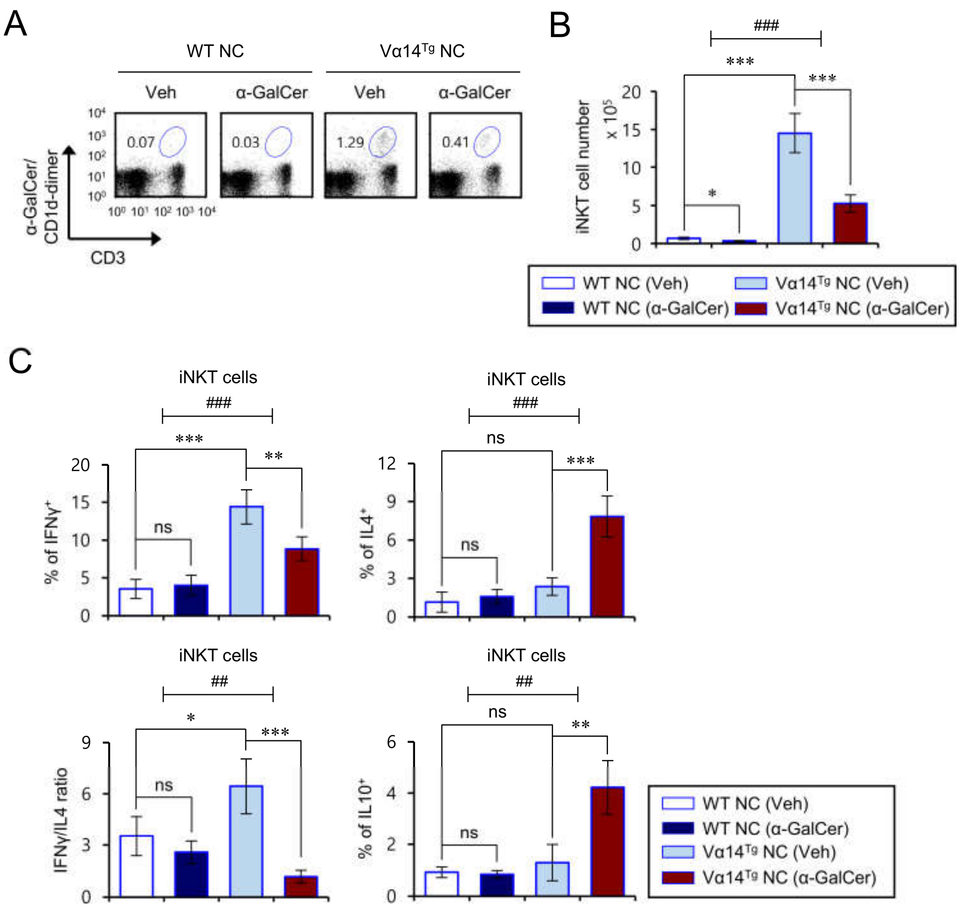

Figure S2. Repeated α-GalCer treatment polarizes Vα14^{Tg} NC iNKT cells toward type 2 cytokine production under SPF conditions.

(A-C) Spleens were prepared from mice as shown in Fig. S1. (A-B) The frequency and absolute cell number of splenic iNKT cells (α-GalCer/CD1d-dimer^{+}CD3^{+}) were determined by flow cytometry. (C) The levels of intracellular IFNγ, IL4, and IL10 were analyzed in splenic iNKT cells (α-GalCer/CD1d-dimer^{+}CD3^{+}). The mean values ± SD (n = 4 in A, B, and C; per group in the experiment; Student's t-test; * $P < 0.05$, ** $P < 0.01$, *** $P < 0.001$) are shown. Two-way ANOVA (Vα14 TCR Tg × α-GalCer) showed an interaction between these two factors ($^{\#\#}$ $P < 0.01$ and $^{\#\#\#}$ $P < 0.001$).
